# A Prospective Study of the Potential Moderating Role of Social Support in Preventing Marginalization Among Individuals Exposed to Bullying and Abuse in Junior High School

**DOI:** 10.1007/s10964-014-0145-4

**Published:** 2014-07-02

**Authors:** Ida Frugård Strøm, Siri Thoresen, Tore Wentzel-Larsen, Åse Sagatun, Grete Dyb

**Affiliations:** 1 Norwegian Centre for Violence and Traumatic Stress Studies, Pb 181 Nydalen, 0409 Oslo, Norway; 2 Centre for Child and Adolescent Mental Health, Eastern and Southern Norway, Pb 181 Nydalen, 0409 Oslo, Norway; 3 Institute of Clinical Medicine, University of Oslo, P.O. Box 1171, 0318 Blindern, Norway

**Keywords:** Abuse, Bullying, Longitudinal, Social support, Use of social welfare benefits, Marginalization

## Abstract

Negative physical and psychological long-term consequences of abuse and bullying are well documented. It is reasonable to assume that abuse and bullying early in life also may have an impact on the ability to work and stay economically independent later in life, but such prospective studies are lacking. This study investigates the consequences of exposure to abuse and bullying in junior high school, as measured by receiving long-term social welfare benefits in young adulthood. In addition, it explores the potential protective role of social support. Self-reported data from 13,633 (50.3 % female) junior high school students were linked to registry data on their use of social welfare benefits from the age of 18 and for eight consecutive years. Cox regression analyses were applied to test the relationship between exposure to life adversities and the use of social welfare benefits, and the potential moderating role of social support. The analyses showed that individuals exposed to abuse and bullying had an increased likelihood of receiving social-welfare benefits compared with individuals not exposed to these types of abuse. Exposure to multiple types of abuse led to a higher likelihood of using social welfare benefits compared with single types of abuse and no abuse. The findings on the potential moderating role of social support were mixed, depending on the source of social support. Family support and classmate relationships were protective in reducing the likelihood of the use of social welfare benefits, whereas peer and teachers’ support showed inconsistent patterns. These results are promising in terms of preventing the long-term negative consequences of abuse and bullying.

## Introduction

Exposure to physical abuse, sexual abuse, and bullying are considered to be major public health problems (Gellert et al. 2010). Although the severe long-term physical (Wegman and Stetler 2009; Annerbäck et al. 2012) and psychological (Turner et al. 2006; Arseneault et al. 2010) health consequences have been documented, few epidemiological studies have focused on the long-term consequences of abuse and bullying during adolescence on reduced participation in work in terms of using social welfare benefits in adulthood (Strøm et al. 2013). Even less attention has been given to potential protective factors, such as social support, in preventing later marginalization among vulnerable youth. A social ecological perspective (Bronfenbrenner 1977) allows us to investigate abuse occurring in multiple areas as well as to examine the interplay between the individual and his or her surroundings. This can help identify possible push and pull factors, such as social support, that may contribute in either a positive or negative way to the individual’s development. It may be important to study exposure to abuse and bullying simultaneously as previous research has found that they often co-occur (Duncan 1999). Children exposed to abuse may lack social abilities and have difficulties with relationships because of their abuse experiences and insecure attachments. As a result, they may be excluded or may become victims of further bullying (Kim and Cicchetti 2010).

Employment is a crucial part of our daily lives. It provides income, skill acquisition, and social connections; it also contributes to one’s self-identity and health, and adds structure to the day (Ross and Mirowsky 1995; Caspi et al. 1998; Tam et al. 2003). On the other hand, reduced participation in work in terms of using social welfare benefits, can be marginalizing. Marginalization occurs when a person is on the “sidelines” of society, moving towards social exclusion, while still having a chance of inclusion (Hyggen and Hammer 2013; Normann 2007). Marginalization may have negative consequences for both the individual and society. At the individual level, marginalization may lead to or exacerbate mental and physical health problems; while at the societal level, it may lead to loss of work productivity, loss of income revenue and taxes, and increased expenses in terms of social welfare benefits and the use of healthcare (Hyggen and Hammer 2013; Ttofi and Farrington 2012; Rasmussen et al. 2010). It is, therefore, a public health problem that needs to be addressed further.

The few studies that have examined reduced participation in work found that individuals who experienced childhood sexual abuse, physical abuse, or both, have a higher likelihood of receiving social welfare benefits (Smith 2005; Derr and Taylor 2004). They also have an increased risk of being fired (Sansone et al. 2012), unemployment, poverty, using Medicaid (Zielinski 2009; Liu et al. 2012), work impairment (Anda et al. 2004; Tam et al. 2003), and income deficits (Mersky and Topitzes 2010). However, these studies were limited by their use of cross-sectional and retrospective designs, a lack of sociodemographic variables, and their reliance on self-reported work performance/status. Only three of these studies were epidemiological investigations (Anda et al. 2004; Liu et al. 2012; Zielinski 2009); the other studies were limited to smaller specified samples of severe cases of abuse (Derr and Taylor 2004; Sansone et al. 2012; Smith 2005; Tam et al. 2003). Two of the studies (Anda et al. 2004; Liu et al. 2012) examined adverse childhood experiences and work impairment/unemployment. Both investigations found a graded relationship between adverse childhood experiences and work impairment in that the unemployment rate and rate of work problems were significantly higher for individuals who reported multiple adverse childhood experiences. Liu et al. (2012) found, in a sample of 17,469 individuals between the ages 18–64, that educational attainment, marital status, and social support mediated the relationship between multiple adverse childhood experiences and unemployment, while Anda et al. (2004) found, in a sample of 9,633 adults, that the relationship between the adverse childhood experiences score and work impairment was mediated by interpersonal relationship problems, emotional distress, somatic symptoms, and substance abuse. Liu et al. (2012) argued that the relationship between adverse childhood experiences and unemployment may be due to the adverse childhood experiences impairing the children’s cognitive ability, which may result in lower educational attainment and social isolation, which, in turn, may reduce the likelihood of employment. The third epidemiological study (Zielinski 2009) found, in a sample of 5,005 individuals between the ages of 18–54, that adults with a history of maltreatment had increased rates of unemployment, poverty, and Medicaid use. On the basis of previous research in various fields, the author suggested that possible pathways between maltreatment and socioeconomic well-being may be educational attainment, psychopathology, and physical health. To the authors’ knowledge, only three previous studies have been conducted on bullying and reduced participation in work. Two studies used small clinical samples, while the third used prospective data to document the relationship between bully victimization and work related outcomes, including: (a) unemployment; (b) having a greater number of different jobs; (c) being paid under the table; (d) having difficulties keeping jobs; and (e) having been fired (Varhama and Björkqvist 2005; Sansone et al. 2013; Wolke et al. 2013). The authors are aware of only one study that focused on both abuse and bullying and later participation in work (Strøm et al. 2013). This study found that exposure to violence and bullying increased the odds of lower participation in work, independent of whether the individuals completed high school.

Social support is beneficial to health and longevity (Thoits 2011) and has been found to have a buffering effect for individuals exposed to stressful events (Cohen and Wills 1985; Thoresen et al. 2014) such as physical abuse, sexual abuse, and bullying. In these cases, social support protects individuals from the potential negative health effects of stressful events (Cohen et al. 2000). Social support encompasses several aspects of social interaction, and an important conceptual distinction is made between received and perceived support. Cohen and Wills (1985) found that the belief that support is available is more important for health and adjustment than support that was actually received. Previous research has shown both mediating and moderating effects of perceived social support on the relationship between abuse and a range of outcomes, including psychopathology (Sperry and Widom 2013) and developmental outcomes (Pepin and Banyard 2006). Similar buffering effects of social support have also been found for bullying and mental health outcomes (Holt and Espelage 2007; Rigby 2000), and student adjustment (Demaray et al. 2005) and well-being (Flaspohler et al. 2009). However, social support may not always have a positive effect in that it may involve modeling risky behaviors by one’s social network, which, in turn, may negatively affect health (Thoits 2011; Gifford-Smith and Brownell 2003). Moreover, Malecki and Demaray (2003) emphasized the importance of distinguishing between the sources of support (e.g., from parents and friends), and the types of support (informational, emotional, appraisal, and instrumental) as the outcome may differ depending on the source of support. Their findings show that support from parents was mostly emotional, informational, and contributed to the student’s personal adjustment. Teachers’ support was mostly informational, but it was their emotional support that contributed to the student’s social skills and academic competence. Classmates and friends provided mostly instrumental support in addition to emotional support. Their research stresses the complexities of social support and how each source of support may be associated with different outcomes. Considering the complexities of social support and that the outcome may differ depending on its source, it is important to distinguish the different sources to determine which one may protect abused adolescents from potentially negative consequences.

Lack of social capital or social ties has also been found to make youth vulnerable to unemployment (Caspi et al. 1998), yet only a scarce amount of research has looked at the protective role of social support on later work-participation outcomes for individuals exposed to life adversities. However, one study on employment found that social support was a protective pathway in the relationship between life adversities and unemployment, while another found that interpersonal problems were a risk factor for work impairment (Liu et al. 2012; Anda et al. 2004).

## The Current Study

Previous research indicates an association between life adversities and later work impairment, and that social support may serve as a protective factor. In this study, we address some of the methodological limitations of previous research by prospectively investigating the potential, long-term consequences of sexual abuse, physical abuse, and bullying—separately and in combination— on receiving long-term social welfare benefits (Aim 1). Furthermore, we explore whether social support moderates this relationship (Aim 2). This constituted two research questions to be examined: (1) Is exposure to abuse and/or bullying in junior high school associated with receiving long-term social welfare benefits in young adulthood? (2) Does social support moderate this relationship? The unique dataset, which combined questionnaire and registry data with a high response rate, allowed us to follow a general population of 13,633 15-year-olds from completion of high school into young adulthood, using objective measures of the use of social welfare benefits.

## Methods

### Procedure

The baseline data are from a large health survey (The Youth Studies) conducted in six counties (Oslo, Hedmark, Oppland, Nordland, Troms, and Finmark) in Norway from 1999 to 2004 by the Norwegian Institute of Public Health and the University of Oslo. The self-report survey was distributed to all 10th graders in the participating counties, and was completed it in the classroom. The participants and their parents received written information regarding the survey prior to completing the questionnaire. They were informed about who was responsible for the survey, the purpose and the content of the survey, how the survey would be conducted, and how the data would be used. The participation was voluntary, and a consent form was signed by the 16-year-olds, while the parents signed for the students who had not turned 16 years at the time of the study (Søgaard and Eide 2005: http://www.fhi.no/eway/default.aspx?pid=240&trg=Content_6671&Main_6664=6898:0:25,7898:1:0:0:::0:0&MainContent_6898=6671:0:25,7899:1:0:0:::0:0&List_6673=6674:0:25,7905:1:0:0:::0:0&Content_6671=6683:91213::1:6682:1:::0:0).

All individuals residing in Norway are registered with a unique personal identification number, which was used to link the questionnaire data with Norwegian registry data from the Historical Event Data Base (FD-Trygd). The FD-Trygd database is managed by Statistics Norway and provides information about social welfare benefits that Norwegian citizens receive. This procedure resulted in a dataset that was used to relate the questionnaire responses for each individual at ages 15–16 to information about their social welfare benefits from age 18 up to the age of 26, depending on the county they were from. The baseline data were collected at different time points for each county (from 1999 to 2004). The follow-up time ended February 2010 and ranged between 4 and 8 years. The study was approved by the Norwegian Institute of Public Health, the Regional Committee for Medical and Health Research Ethics, and by the Norwegian Data Inspectorate.

### Participants

All registered 10th graders in the six counties were invited (n = 18,455) to participate in the baseline studies. Of the invited students, 15,966 (87 %) participated and 14,063 (88 %) agreed to linking the data. Not all respondents answered whether they had been exposed to abuse or bullying (n = 430). Thus, the sample comprised 13,633 individuals, which constitutes a response rate of 73.9 % of the invited students. In this investigation, adolescence is defined as ranging from ages 16–18, while young adults are defined as 18–26 years. The term “young adult” has no set definition. However, it is a period that is distinct from childhood, adolescence, and adulthood, and is characterized by finding one´s identity and deciding their life path. It is a time when a majority of individuals leave their parents’ home to get established on their own in the transition from adolescence to adulthood (Hyggen and Hammer 2013).

### Measures

#### Social Welfare Benefits

The amount of “time to receive any form of social welfare benefits” was based on registry data from the FD-Trygd database. In order to be registered as receiving social welfare benefits, different cut-off criteria were set for each of the benefits. The social welfare benefits included (a) social assistance (at least 180 days in a year); (b) unemployment (180 consecutive days in a year); (c) sickness benefits (at least 180 days of 100 % unemployment in a year); and (d) a registered rehabilitation allowance, a temporary disability benefit, a disability benefit, and a vocational rehabilitation allowance. If a person met any of these criteria, he/she was registered with an event in the survival analysis (for more details, see “Appendix 1”). As opposed to the event of interest, censored cases terminate observation without occurrence of the event being studied. In this study, individuals who emigrated or died during the study period were censored at the time of death or emigration, and individuals who did not receive any social welfare benefits during follow-up were censored at the end of follow-up.

#### Exposure Variables

##### Sexual Abuse

Exposure to sexual abuse was measured by asking the respondents one question: whether they had experienced sexual abuse within the past 12 months (e.g., indecent exposure, touching, involuntary intercourse), with a dichotomous response format (yes or no).

##### Physical Abuse/Violence

Exposure to violence was measured by asking the respondents whether they had been exposed to any violence within the past 12 months (e.g., had been punched, kicked, or similar events), with the response options of never, yes by youths, yes by adults, or yes by both youths and adults.

##### Bullying in School

Experiences of bullying were measured by asking the respondents one question: whether they had experienced problems with bullying in school, or on the way to or from school during the past 12 months. The response format was: never (1), sometimes (2), about once a week (3), and many times a week (4). To investigate the association between the outcome variable and each type of abuse separately and in combination, a variable was created that collapsed across the response options. Thus, the categories were: (1) not exposed to any abuse; (2) exposed to bullying “only;” (3) exposed to violence “only;” (4) exposed to sexual abuse “only;” and 5) exposed to two to three types of abuse (a combination of bullying and/or violence and/or sexual abuse).

#### Sociodemographic Variables

Each respondent’s gender, age, perceived financial situation, living situation, parents’ birthplace, marital status, education level, and employment at baseline were included in the models to adjust for sociodemographic differences.

##### Perceived Financial Situation

The adolescents were asked to report whether one’s family, in comparison with other families in Norway, had “poor,” “somewhat good,” “good,” or “very good” finances.

##### Living Situation

The respondents were asked if they lived with: “mother and father,” “just mother,” “just father,” “the same amount with mother and father separately,” “mother or father and a new partner,” “foster parent,” or “other.”

##### Parents’ Birthplace

The parent’s birthplace was registered by asking whether the mother and father were born in Norway or in another country. If they were born in another country, the respondent was asked to list the country. This resulted in responses for 13 countries, which were recoded into three categories: Norway (at least one parent from Norway), Western countries (except Norway, with at least one of the parents being from Western Europe, North America, or Australia), and non-Western countries (both parents being non-Western). This categorization is in agreement with Statistics Norway’s definition of Norwegian ethnicity where at least one of the parents needs to be Norwegian in order for the parents to be classified as Norwegian.

##### Parents’ Marital Status

The parents’ marital status was reported by asking if the parents were “married,” “unmarried,” “divorced/separated,” “one or both dead,” or “other.”

##### Parents’ Employment

The adolescents’ were asked to report whether their father and/or mother were currently working, with the employment responses being “full-time work,” “part-time work,” “unemployed/on welfare,” “stays at home,” “goes to school/studies,” and “deceased.”

##### Parents’ Education Level

Parents’ education was collected from the National Education registry data and categorized as “highest level of education” (more than 4 years), “high level of education” (up to 4 years), “high school,” “junior high school,” or “unregistered.”

#### Social Support Variables

Mean scores were calculated for each scale of four items from respondents who answered at least two items. For the family support scale, three of the five items had to be answered to be included. All items had a response format on a scale of 1 (strongly agree) to 4 (strongly disagree). The mean scores were reversed so that a score of 4 indicated strong perceived support. The social support variables were assessed at baseline.

##### Teachers’ Support

The measure of teacher’s support included four items: (1) my teachers appreciate my opinions; (2) my teachers appreciate me; (3) my teachers help me with my subjects when I need it; and (4) my teachers help me with my personal problems if needed. Cronbach’s alpha was 0.81.

##### Classmate Relationships

The measure of classmate relationships included four items: (1) I like my classmates; (2) I have lots in common with my classmates; (3) I feel attached to my classmates; and (4) my classmates value my opinions. Cronbach’s alpha was 0.83.

##### Family Support

The family support measure included five items: When you think about your family, would you say that: (1) I feel attached to my family; (2) my family takes me seriously; (3) my family values my opinions; (4) I mean a lot to my family; and (5) I can count on my family when I need help. Cronbach’s alpha for this scale was 0.86.

##### Friends’ Support

The measure of friend’s support included four items: When you think about your friends, would you say that: (1) I feel closely attached to my friends; (2) my friends value my opinions; (3) I can help/support my friends; and (4) I can count on my friends when I need help. Cronbach’s alpha for this scale was 0.83.

### Statistical Methods

Chi square tests were used to examine associations between exposure and abuse, bullying, and demographics. Analysis of variance was used to compare social support for the exposed and non-exposed. Kaplan–Meier analyses were used to compare the time to receive social welfare benefits for the non-exposed and the different exposure groups, using the whole cohort. Cox proportional hazard regression was used to test relationships of abuse and bullying in junior high school with the use of social welfare benefits and also to examine whether social support served as a protective factor against receiving social welfare benefits in young adulthood among those exposed. In Cox regression, the interpretable information stated for each covariate is given in terms of hazard ratios, ratios between instantaneous risks for the event being studied. The proportional hazard assumption is that the hazard ratios are the same throughout follow-up. First, univariate relationships between exposure and time to receiving social welfare benefits were tested. This was followed by hierarchical Cox regression, in which exposure to abuse and bullying, friends’ support, family support, teachers’ support, and classmate relationships were first (model I), followed by sociodemographic characteristics (model II). Bootstrap analysis was conducted to test for significant differences between the hazard ratios for exposure in unadjusted analyses and model I. In model III, interactions between exposure and the social support scales were included. Model III was also used to study the effect of each social support scale within each of the exposure groups. Missing data from the included variables in the model were removed from the Cox regression analyses. In all Cox regression analyses, ties were handled by the Efron procedure. The proportional hazard assumption was checked, as described in Therneau and Grambsch (2000). First, a global *p* value for deviations from the proportional hazard assumption is computed for the model as a whole. If significant, *p* values for deviations from proportional hazard for individual hazard ratios should be further investigated by a graphical procedure using smoothed plots of Schoenfeld residuals, including confidence bands. If substantial deviations from proportional hazard are detected, one possibility is to run separate Cox regression analyses in different parts of the follow-up time. In this case, investigations, when considered necessary, were done only for the social support and exposure variables. Specifically, in case of significant and substantial deviations from the proportional hazard assumption, separate analyses were conducted within two time periods determined from inspection of the plots of the Schoenfeld residuals intervals (<2 years and ≥2 years). The R (The R Foundation for Statistical Computing, Vienna, Austria) packages rms and boot were used for Cox regression, bootstrapping, and testing the proportional hazard assumption. PASW Statistics 18 (formerly SPSS Statistics 18, IBM) was used for all other analyses.

## Results

### Demographics

The respondents were 16 years old at baseline, 18 years old when the follow-up started, and 22–26 years old at the end of follow-up. Overall, the individuals exposed to abuse reported somewhat more disadvantageous sociodemographic characteristics compared with the individuals not exposed to abuse (Table 1). However, the major trends remained the same for both groups. The majority of the respondents reported a somewhat good or good financial situation. Most had Norwegian parents who were married, and more than half of the sample lived with both parents, although a substantial proportion had divorced parents. Most of the adolescents had a father who worked full-time and a mother who worked either part-time or full-time. Finally, the majority of the parents had completed high school as their highest level of education, and about one-third of the parents had some higher education (i.e., completed at least 16 years of education).

**Table 1:** Sociodemographic characteristics of individuals exposed to abuse compared to the non-abused individuals (Total *n* = 13,633)

Sociodemographic characteristics	Individuals not exposed to abuse or bullying (%)	Individuals exposed to abuse or bullying (%)
Gender		
Female	52.5 (5,016)	45.2 (1,844)
Male	47.5 (4,525)	54.8 (2,238)
Perceived financial situation		
Poor	2.5 (239)	5.0 (203)
Somewhat good	31.5 (2,976)	37.1 (1,497)
Good	56.4 (5,333)	48.6 (1,961)
Very good	9.6 (911)	9.4 (378)
Parents’ marital status		
Married/cohabitants	69.5 (6,622)	60.8 (2,471)
Unmarried	3.3 (312)	4.1 (166)
Divorced/separated	22.6 (2,158)	29.3 (1,192)
One or both dead	2.8 (269)	3.3 (136)
Other	1.8 (167)	2.5 (100)
Parents’ employment—Father		
Yes. full-time	82.4 (7,674)	77.3 (3,041)
Yes. part-time	6.8 (633)	8.0 (315)
Unemployed/on welfare	5.3 (498)	7.3 (288)
Stays at home	2.4 (225)	3.2 (126)
Goes to school/study	1.1 (104)	1.4 (55)
Dead	1.9 (180)	2.8 (111)
Parents’ employment—Mother		
Yes. full-time	59.3 (5,571)	56.2 (2,247)
Yes. part-time	21.7 (2,041)	21.7 (866)
Unemployed/on welfare	5.1 (479)	7.0 (280)
Stays at home	9.6 (905)	10.1 (405)
Goes to school/study	3.4 (318)	3.9 (155)
Dead	0.8 (77)	1.1 (43)
Parents’ education		
Highest level of education (>4 years)	14.5 (1,382)	12.9 (525)
High level of education (≤4 years)	31.6 (3,007)	29.2 (1,191)
High school	40.3 (3,841)	41.7 (1,700)
Junior high school	12.5 (1,191)	14.9 (608)
Unregistered	1.1 (108)	1.2 (49)
Parents’ birthplace		
Norway	88.5 (8,401)	87.4 (3,538)
Western country	0.9 (81)	0.9 (35)
Non-Western country	10.6 (1,011)	11.8 (477)
Living situation		
Mother and father	70.7 (6,737)	61.6 (2,509)
Just mother	12.7 (1,210)	16.1 (655)
Just father	2.5 (242)	3.4 (137)
The same time with mother and father	4.8 (458)	5.7 (233)
Mother or father and new partner	8.0 (764)	10.3 (419)
Foster parents	0.5 (47)	1.3 (51)
Other	0.7 (66)	1.7 (71)

Not all of the respondents completely reported the sociodemographic items. The n varies for each item (missing values ranges from 0.1 to 2.8 %)

Gender (χ^2^ = 61.70, *df* = 1, *p* < .001). Perceived financial situation (χ^2^ = 113.11, *df* = 3, *p* < .001). Parents marital status (χ^2^ = 99.01 *df* = 4, *p* < .001). Parents employment—father (χ^2^ = 50.93, *df* = 5, *p* < .001). Parents employment—mother (χ^2^ = 27.25, *df* = 5, *p* < .001). Parents education (χ^2^ = 24.53, *df* = 4, *p* < .001)

Parents birthplace (χ^2^ = 3.71, *df* = 2, *p*. 157). Living situation (χ^2^ = 141.14, *df* = 6, *p* < .001)

### Prevalence of Exposure to Life Adversities and the Use of Social Welfare Benefits

Most of the respondents were not exposed to any abuse (n = 9,551, 70.1 %). The most frequent type of abuse was violence only (n = 1,793, 13.2 %), followed by bullying only (n = 1,141, 8.4 %), two to three types of abuse (n = 902, 6.6 %), and sexual abuse only (n = 246, 1.8 %). More than half (n = 282, 53.4 %) of all individuals exposed to sexual abuse (n = 528) had also been exposed to another type of abuse.

**Fig. 1:**
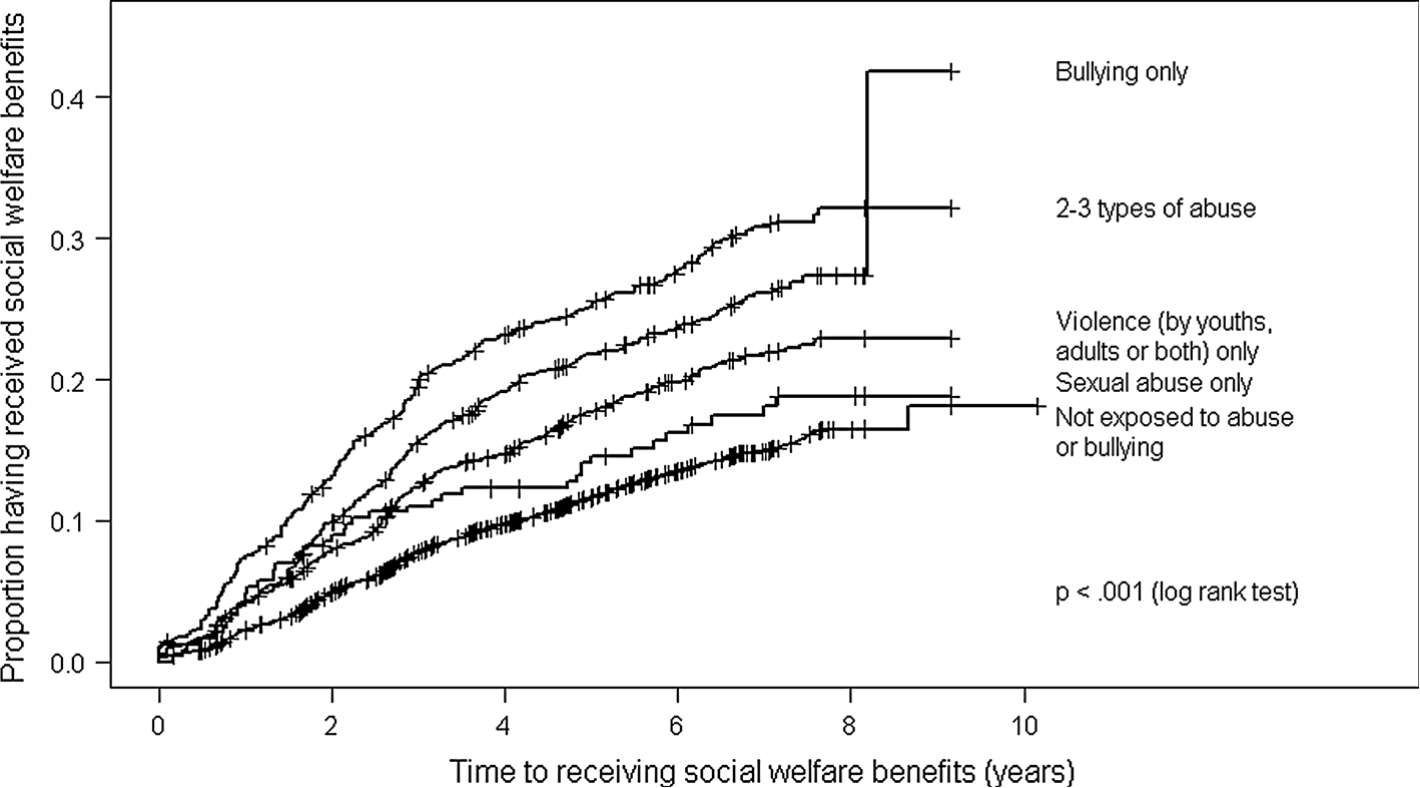
Proportion of having received social welfare benefits (N = 13, 576)

Of the total sample, 16.7 % (2,273) received some form of social welfare benefits, while the rest of the sample was not registered as having an event during the follow-up period (n = 11,303). Individuals who were not exposed to abuse in junior high school had a lower likelihood (n = 1,324, 13.9 %) of receiving social welfare benefits compared with individuals exposed to abuse (Fig. 1). Individuals exposed to two to three types of abuse (any combination of bullying, violence, or sexual abuse) had the highest risk (n = 262, 29 %) of receiving social welfare benefits, followed by individuals exposed to bullying only (n = 279, 24.5 %), violence only (n = 366, 20.4 %), and sexual abuse only (n = 42, 17.1 %).

### Exposure to Life Adversities and Social Support

The exposed group reported lower levels of social support compared with the non-exposed group. However, levels of perceived support from friends and family were generally high, with a mean level above 3 on a scale from 1 to 4, where 4 shows high support. Teacher and classmate relationships had a somewhat lower rating with a mean below 3 for the exposed group and 3 for the non-exposed group. The social support scales were moderately but significantly correlated with each other, with the highest correlation being between classmate relationships and teachers’ support (r = .455, *p* < .001).

### Aim 1: Relationships Between Exposure to Abuse and Bullying in Junior High School and Later Usage of Long-Term Social Welfare Benefits

The first column of Table 2 displays the results of the univariate Cox regression analyses of the likelihood of receiving long-term social welfare benefits for each of the exposed groups compared with the non-exposed group. All of the exposed groups, except the sexual abuse only group, had a higher likelihood of receiving long-term social welfare benefits compared with the non-exposed adolescents. Individuals exposed to two to three types of abuse had twice the hazard of receiving social welfare benefits, followed by individuals exposed to bullying only, who had a 90 % higher hazard of receiving benefits compared with the non-exposed individuals.

**Table 2:** Cox regression analyses of the relationship between exposure and receiving social welfare benefits

	Unadjusted HR (95 % CI); *p* value	Model I: Adjusted for social support HR (95 % CI); *p* value	Model II: Adjusted for social support and sociodemographic characteristics HR (95 % CI); *p* value
Not exposed to abuse or bullying^a^			
Bullying only	1.90 (1.65, 2.19); < .001	1.66 (1.44–1.92); < .001	1.59 (1.37–1.83); < .001
Violence only	1.52 (1.34, 1.72); < .001	1.35 (1.18, 1.53); < .001	1.27 (1.12, 1.44); < .001
Sexual abuse only	1.23 (.89, 1.71); .213	1.12 (.81, 1.55); .504	1.15 (.83, 1.60); .403
2–3 types of abuse	2.26 (1.95, 2.61); < .001	1.66 (1.42, 1.94); < .001	1.47 (1.26, 1.72); < .001

Unadjusted and adjusted model, adjustment for social support (model I). Gender, age, economic situation, parents’ education, parents’ birthplace, parents’ marital situation, father’s employment, mother’s employment, and living situation (model II)

^a^Reference category. *P* value for model II (χ^2^ (30 *n* = 12 547) = 822.68 *p* = < .001)

### Aim 2: Social Support as a Moderator Between Exposure to Abuse and Bullying in Junior High School and Later Usage of Long-Term Social Welfare Benefits

In model 1 (Table 2), when the social support scales were added to the model, the pattern remained the same, but a reduction in the hazard ratios was observed. The hazard ratio for bullied youth was reduced by 24 % (reduction from hazard ratio 1.90–1.66), and there was a similar reduction by 17 % for victims of violence, and by 60 % for individuals exposed to two to three types of abuse. The bootstrap analyses showed that the hazard ratio for bullying only (ratio 1.66/1.90 = .87, CI .84–.91), violence only (ratio 1.35/1.52 = .89, CI .86–.91), and two to three types of abuse (ratio 1.66/2.26 = .74, CI .69–.78) was significantly reduced when social support was added to the model. The hazard ratios were further reduced for each of the significant exposure categories when sociodemographic data were added to the model (model II). In model III (not included in Table 2), the interactions between the four social support scales and exposure taken together were significant (all interactions, χ^2^ (16) = 28.19, *p* = . 030). However, when each interaction was examined individually, only the interaction between friends’ support and exposure was close to statistical significance (*p* = .056).

To explore the findings presented in Table 2 further, the effect of each source of social support within each of the exposure groups was examined (Table 3). The analysis showed that family support and classmate relationships led to a reduced likelihood of receiving long-term social welfare benefits, in that the hazards ratios within each of the exposure groups were below 1. The results for teachers’ support showed the same pattern, with the exception of teachers’ support within the sexual abuse only group. Finally, friends’ support showed an inconsistent pattern, in which victims of bullying only, violence only, and individuals exposed to two or more types of abuse had an increased risk of receiving social welfare benefits at higher levels of friends’ support.

**Table 3:** Cox regression analyses of social support within each exposure group and receiving social welfare benefits

	Not exposed to abuse HR (95 % CI); *p* value	Bullying HR (95 % CI); *p* value	Violence HR (95 % CI); *p* value	Sexual abuse HR (95 % CI); *p* value	Two or more types of abuse HR (95 % CI); *p* value	Interactions^a^
Friends’ support	.98 (.86–1.11); .750	1.17 (.92–1.51); .202	1.18 (.92–1.50); .185	.87 (.53–1.41); .561	1.42 (1.12–1.80); .004	.056
Family support	.87 (.78–.97); .012	.77 (.60–.97); .030	.89 (75–1.06); .196	.65 (.40–1.06); .087	.88 (.73–1.05); .148	.675
Teacher’s support	.97 (.88–1.07); .531	.98 (.81–1.18); .810	.85 (.73–.99); .041	1.71 (1.05–2.80); .031	.93 (.78–1.10); .389	.098
Class-mate relationship	.77 (.70–.85); < .001	.99 (.82–1.20); .925	.80 (.68–.95); .011	.72 (.46–1.13); .151	.84 (.70–1.01); .064	.216

Adjustment for gender, age, economic situation, parents’ education, parents’ birthplace, parents’ marital situation, father’s employment, mother’s employment, and living situation

^a^ *p* value for interaction between each social support variable and exposure. Overall *p* value for (all interactions, χ^2^ (16) = 28.19, *p* = . 030). Centered values for social support scores, for use in interaction models, were chosen based on histograms: Friends’ support: 3.6. Family support: 3.61. Teachers’ support: 3. Classmate relationships: 3.06

### Testing the Proportional Hazard Assumption

The tests of the proportional hazard assumption indicated global deviations and significant deviations for some of the predictors in the univariate model and in models I and II (see “Appendix 2”). Because of the statistically significant deviations from the proportional hazard assumption, especially for violence only and two to three types of abuse, separate analyses were conducted within different time intervals (<2 years and ≥2 years). These analyses showed that compared with the non-exposed individuals, the individuals exposed to abuse and bullying had a higher likelihood of receiving long-term social welfare benefits during the first 2 years of follow-up, and that the likelihood decreased somewhat after 2 years. Therefore, the corresponding hazard ratios from these analyses are likely to be conservative estimates. Table 4 shows the hazard ratios for exposure in model I in separate analyses, wherein the two time-periods were included. The table shows that the sexual abuse only group, compared with the no exposure group, had a higher likelihood of receiving social welfare benefits during the first 2 years of follow-up, but that this effect was no longer significant afterwards. Individuals exposed to bullying only, violence only, and two to three types of abuse were likely to receive social welfare benefits throughout the follow-up period, although the likelihood was somewhat reduced after 2 years.

**Table 4:** Cox regression analyses (in model I) of exposure and receiving social welfare benefits, including hazard ratios separately for the two first years of follow up and after 2 years of follow up time

	Until 2 years HR (95 % CI); *p* value	After 2 years HR (95 % CI); *p* value
Not exposed to abuse or bullying^a^		
Bullying only	1.80 (1.42–2.27); < .001	1.59 (1.33–1.92); < .001
Violence only	1.55 (1.26–1.91); < .001	1.24 (1.06–1.46); .008
Sexual abuse only	1.62 (1.01–2.60); .047	.863 (.55–1.36); .525
2–3 types of abuse	2.09 (1.65–2.65); < .001	1.42 (1.17–1.75); .001

^a^Reference category. Adjusted for social support

## Discussion

The negative consequences of exposure to abuse and bullying have been well documented. Our study adds to this literature by prospectively investigating their long-term consequences in terms of the later use of social welfare benefits in a large sample, using a unique data registry. The study’s first aim was to prospectively investigate whether abused and bullied adolescents had a higher likelihood of receiving long-term social welfare benefits compared with non-abused adolescents. Our results confirmed this expectation, indicating that individuals who have been abused or bullied are more vulnerable to struggles with participation in work in young adulthood, which aligns with previous research findings (Sansone et al. 2012, 2013; Smith 2005; Mersky and Topitzes 2010; Anda et al. 2004; Varhama and Björkqvist 2005; Zielinski 2009; Wolke et al. 2013). This outcome remained true after controlling for other factors known to have an impact on receiving social welfare benefits, such as parents’ employment, education, and living situation (Hammer 2007). The likelihood was highest during the first 2 years after high school and decreased over time. The first years after high school mark a crucial developmental period from adolescence to adulthood. It is a time when many adolescents leave their parents’ home to become established on their own, find their identity and decide their life path (Hyggen and Hammer 2013). Research has shown that this period is associated with the highest risk of marginalization (Sletten and Hyggen 2013), consistent with stronger hazard ratios of receiving long-term social welfare benefits in this period. As reported by previous research, individuals who are exposed to abuse or bullying have an increased likelihood of having poor health and difficulties with social relationships, making them more vulnerable during the transition from adolescence to adulthood.

Furthermore, a cumulative effect of exposure was observed in our sample, in which individuals exposed to multiple types of abuse had a higher likelihood of receiving long-term social welfare benefits compared with the other groups that were studied. Studies on the health effects of abuse have shown that individuals exposed to more than one type of abuse have more severe health effects compared with individuals exposed to one type of abuse (Finkelhor et al. 2007). This research may help explain why exposure to multiple types of abuse may lead to using more social welfare benefits. Among the single exposure groups, individuals exposed to bullying showed the highest and most consistent likelihood of receiving welfare benefits throughout the study period. This emphasizes the severe long-term consequences of bullying and why it should be regarded as seriously as other forms of abuse. Individuals exposed to sexual abuse only were likely to receive social welfare benefits during the first 2 years after high school, but the likelihood decreased as time went by. This can be explained by the small percentage of individuals being exposed to sexual abuse only. More than half of the individuals exposed to sexual abuse had also been exposed to another type of abuse. Thus, the sexual abuse only group may differ from the groups found in clinical studies. This does not mean that exposure to sexual abuse has less severe consequences than other types of abuse, but rather that sexual abuse victims have a higher likelihood of being exposed to other life adversities, and that this cumulative effect may have a severe impact, such as a higher likelihood of welfare dependency. Moreover, the weak association with receiving social welfare benefits may also reflect a greater variation in the responses to sexual abuse in large epidemiological studies, which include less severe assaults, such as indecent exposure.

The second aim of the study was to test whether social support would moderate the relationship between abuse/bullying and the likelihood of receiving long-term social welfare benefits. Although a large volume of research has documented the positive effect of social support, few studies have attempted to explain the possible pathways that may protect abused or bullied adolescents from later marginalization. However, some studies have stressed the importance of social relationships for vulnerable groups as a protective factor in preventing later unemployment and as a risk factor for work impairment if one has relationship problems (Liu et al. 2012; Anda et al. 2004). Our results partly support these findings. The initial analyses showed that social support moderated the reduction of the likelihood of receiving social welfare benefits for individuals exposed to violence, bullying, and multiple types of abuse. However, the results were mixed when the interaction between social support and abuse and bullying were investigated. Family support and positive classmate relationships served as a protective factor for all the exposure groups, which is consistent with findings that show positive benefits associated with having these relationships. Both family support and positive classmate relationships may contribute to higher self-esteem, psychological well-being, healthy relationships, and school connectedness (Bolger and Patterson 2003; De Ridder et al. 2012; Gallagher 2012; Sapouna and Wolke 2013; Wentzel 1998; Thompson et al. 2006), which, in turn, may improve overall health and reduce the need for using social welfare benefits. It has also been shown that family and social networks are important for work integration, with respect to establishing contacts in the labor market and getting information about potential jobs (Tovatt 2013; Sletten and Hyggen 2013). However, these mechanisms are not well understood and need to be investigated further.

Moreover, our results found an inconsistent pattern of friends’ support. This may be explained by the fact that abused and bullied adolescents may have a higher likelihood of displaying antisocial behavior (Smith et al. 2005). They may therefore socialize with friends who model external and risky behavior, which, in turn, may lead to negative rather than positive outcomes (Thoits 2011; Gifford-Smith and Brownell 2003; Bender 2010; Gifford-Smith and Brownell 2003). Also, some of the bullying research has shown that friendship alone cannot protect the individual from some of the negative consequences of bullying, but having fewer friends can lead to lower levels of delinquency (Sapouna and Wolke 2013; Rothon et al. 2011; Pouwelse et al. 2011). A possible explanation for the small effect of teachers’ support in the current study may be that the social environment promoted by the teacher is reflected in the classmate relationships, rather than in teachers’ support itself. These findings emphasize the importance of studying each source of social support separately to determine their individual effects on exposure and receiving social welfare benefits.

### Strengths and Limitations

The baseline data were self-reported and no other data were gathered from other potential informants, such as parents or teachers. This might have provided a more accurate number of possible exposures to abuse and bullying. The measures had some weaknesses in that: (a) the severity or chronicity of the exposure was not specified; (b) it was not reported who committed the abuse (other than adults or youths for physical abuse); and (c) the abuse was limited to physical and sexual abuse. As the different bullying roles were not specified, the victim could have been a bully and/or a victim. Previous studies have shown that the use of specific, behaviorally formulated questions reduces false negative responses and obtains higher prevalence rates compared with labelling questions, especially when dealing with sensitive and stigmatized issues, such as rape (Harned 2004). The use of simple questions might therefore have led to an underestimation of the exposure. This, in turn, may have affected the relationships with the outcomes since exposed adolescents have a higher likelihood of marginalization compared with non-exposed adolescents. Furthermore, the two phenomena may occur in the same situation, such as physical abuse and bullying; therefore, it is uncertain whether respondents who answered affirmatively to both questions experienced one or two incidents.

The registry database was of good quality with few missing data. However, the prevalence of social welfare dependency may vary somewhat according to the cut-offs imposed for the duration of receiving social welfare benefits. The cut-offs were set to only include the long-term use of social welfare benefits, and similar cut-offs to those used in other studies were applied (De Ridder et al. 2012; Normann 2007). Not all individuals who receive some form of social welfare benefit are likely to be marginalized, and they may return to work. However, this may depend on the type of social welfare benefit received. Research has also shown that the likelihood of recurrent use of social welfare benefits is greater among individuals who have previously received benefits or have been out of work for a longer period of time (Raaum et al. 2009; Hyggen and Hammer 2013).

Attrition cannot be excluded. Not all participants consented to linkage with registry data and there were some missing values in the variables used. Thus, our analyses are based on 73.9 % of the invited 10th graders in the respective counties for the years in question. A study using the same data found that 12 % of the participants in the baseline study did not consent to linkage with registry data. However, they did not differ significantly in the gender distribution or in the report of mental health problems (Sagatun et al. 2014). Another study, partly based on the same data, considered response rates and selection problems by investigating mental health and health behavior variables. Here, the association measures (prevalence ratios) were quite similar among participants and all invitees (Bjertness et al. 2010). In this study, the response rate was quite high, and, thus, the findings are expected to be fairly representative of the study population.

The high response rate is an important strength of the study, in addition to the large population-based sample and its longitudinal design. The registry data allowed us to follow a large group of adolescents and their use of social welfare benefits for up to eight consecutive years. These data are unique to the Scandinavian countries and provide an extraordinary opportunity to conduct longitudinal studies without burdening the respondents. The current sample was gathered from six counties in Norway and is, therefore, considered to be fairly representative of the country. In addition, linking the registry data to the questionnaire data allowed us to investigate recent reports of abuse (within the past 12 months), rather than using retrospective data, which can result in recall bias (Wegman and Stetler 2009). The self-reported questionnaire also allowed us to investigate perceived social support, which is a valuable asset when studying its effects (Cohen and Wills 1985). To our knowledge, this study is the first to investigate the combined influence of exposure to physical abuse, sexual abuse, and bullying during adolescence and later use of social welfare benefits, in addition to looking at social support as a possible protective pathway in preventing later marginalization.

### Implications

This study demonstrates the serious negative long-term consequences of exposure to abuse and bullying. Thus, preventive efforts in schools to help individuals exposed to abuse and bullying should be emphasized. More research studying exposure to both abuse and bullying are needed. More specifically, risk factors for exposure to bullying among abused children should be investigated along with the outcomes associated with exposure to these types of abuse. Our research shows that individuals exposed to both abuse and bullying have the highest risk of marginalization. This knowledge must be taken into account when planning preventive measures against becoming marginalized. Furthermore, this study points to social support as an important protective factor in preventing later marginalization. The results confirm the complexities of social support and the importance of investigating the associations of the different sources of social support and the outcome. Enhancing a person’s social support network may not be efficient if the friend-network provides a negative influence. The results of the study highlight the importance of strengthening family support and improving classmate relationships for vulnerable groups in order to prevent marginalization. Finally, more research is needed to investigate the mechanisms of social support in preventing later marginalization.

## Conclusion

This study is an important contributor to research on adolescence as it shows that the effects of exposure to abuse and bullying last well beyond junior high school and into young adulthood. Specifically, exposure to physical abuse, sexual abuse, and bullying all predicted later use of social welfare benefits This emphasizes the importance of detecting abuse at an early age so that the negative consequences associated with abuse may be reduced. Moreover, most research studying adolescence and marginalization has focused on loss of education as a pathway to being marginalized (Falch and Nyhus 2011). However, this study stresses the importance of social support and the significance of studying its different sources. Individuals who were exposed to either abuse or bullying or both in junior high school and who had support from classmates or family were less likely to become marginalized compared to exposed adolescence without such support. This indicates that more effort is required to build strong social support networks in junior high school and that there is a need to study different sources of social support and their role in preventing marginalization of vulnerable youth.

## Appendix 1

See Table 5.

**Table 5:** Benefits included in the study, their definitions and the cut-off used in the analyses

Social welfare benefits	Description	Cut-off used in analyses
Sickness benefit	Sickness benefits compensate for loss of income for employed members of the National Insurance Scheme who are occupationally disabled due to an illness or injury	<180 days of 100 % unemployment in a year
Social assistance	Financial assistance is intended to ensure that everyone has enough money to cover their basic subsistence costs. Financial assistance is intended to secure people’s income on a temporary basis and therefore aims to help you become financially independent	At least 180 days in a year
Unemployed	Unemployment benefits are a partial replacement for lost earnings. In order to receive unemployment benefits you must register with the Norwegian Labour and Welfare Administration (NAV) as a jobseeker and actually apply for work in addition to meeting the further requirements	<180 consecutive days in a year
Rehabilitation allowance	Rehabilitation allowance is provided when a person’s work ability is reduced by sickness or injury, but is under treatment with the purpose of returning to work	Registered allowance
Temporarily disability benefit	Temporarily disability benefit is provided for chronically reduced work ability caused by sickness or injury, but with a possibility for improvement of sickness and work ability	Registered benefit
Disability benefit	Disability pension may be relevant for those with permanently impaired earning capacity due to illness or injury	Registered benefit
Vocational rehabilitation allowance	Occupational rehabilitation is an individualized measure that aims to improve your work capabilities and that provides a more extensive placement assistance and guidance than what the Norwegian Labour and Welfare Administration (NAV) offers. Through occupational rehabilitation measures, you can receive help in tackling problems that prevent you from participating in the labour market	Registered allowance

All of the definitions are taken from the NAV (https://www.nav.no/English/English/Information+about+NAV%27s+services+and+benefits.155652.cms, 2,012)

## Appendix 2: Tests of the Proportional Hazard Assumption

### Univariable Analysis for Exposure Categories

The tests of the proportional hazard assumption in the univariable analysis by exposure indicated a deviation (global *p* = .002), particularly for violence (*p* = .007) and multiple exposure types (*p* < .001) compared with no exposure. The estimate was within or at the confidence bands, although decreasing effects could be noticed.

### Univariable Analysis for Social Support Variables

In univariable models by social support variables there were significant deviations for family (*p* = .010) and classmate (*p* = .012) support.

### Models 1 and 2 for Exposure Categories

In models 1 and 2 (global *p* < .001) there were significant deviations for violence (*p* = .011 and .034) and multiple exposure types in model 1 (*p* = .019) compared with no exposure.

### Models 1 and 2 for Social Support Variables

In both models 1 and 2, there was significant deviations for teacher support (*p* = .007 and .006). In both models, the estimate was above the confidence bands at later times where the plot was consistent with no effect. Generally the plots were consistent with a gradual decrease in the effects and no substantial effects from about 2 years.

### Model 3

For model 3 there was a significant deviation (global *p* < .001), but no significant deviations for any interaction (*p* ≥ .055).
